# Greening the Solid-Phase Peptide Synthesis of the First Bicyclic Analogue of the Arc Repressor and Its Binding to DNA

**DOI:** 10.1021/acs.joc.5c01902

**Published:** 2025-11-11

**Authors:** Eleonora Procino, David Bouzada, Sara D’Ingiullo, Lorenza Marinaccio, Igor Zhukov, Azzurra Stefanucci, Adriano Mollica

**Affiliations:** † Department of Pharmacy, University of Chieti-Pescara “G. d’Annunzio”, Via dei Vestini 31, 66100 Chieti, Italy; ‡ Centro Singular de Investigación en Química Biolóxica e Materiais Moleculares (CiQUS), Departamento de Química Orgánica, 430234 Universidade de Santiago de Compostela, 15782 Santiago de Compostela, Spain; § Department of Innovative Technologies in Medicine and Dentistry, University “G. d’Annunzio” of Chieti-Pescara, Via dei Vestini, 66100 Chieti, Italy; ∥ Laboratory of Biological NMR, Institute of Biochemistry and Biophysics, 90863 Polish Academy of Sciences, ul. Pawińskiego 5A, 02-106 Warsaw, Poland

## Abstract

Biological macromolecules such as proteins often interact with double-stranded DNA through the formation of hydrogen bonds in grooves, thereby modulating the accessibility of transcription factors to specific DNA sequences. Since the primary sequence of the arc repressor responsible for DNA binding and the associated 3D conformational requirements are well characterized, we have designed and synthesized a bicyclic analogue by ultrasound-assisted solid-phase peptide synthesis using a green approach. This new molecular entity was characterized by circular dichroism to verify the β-turn conformation and by a series of NMR experiments to elucidate its 3D structure. Its interaction with DNA oligomers containing the TAGA box was evaluated by using a battery of DNA displacement assays. Fluorescence quenching experiments revealed the close proximity between tryptophan residues and DNA bases, supporting the peptide–DNA interaction. Overall, the data demonstrate that this bicyclic β-sheet arc mimetic engages DNA with sequence selectivity, and to our knowledge, this represents the first report of such a design exhibiting topological preference for DNA grooves.

## Graphical abstract

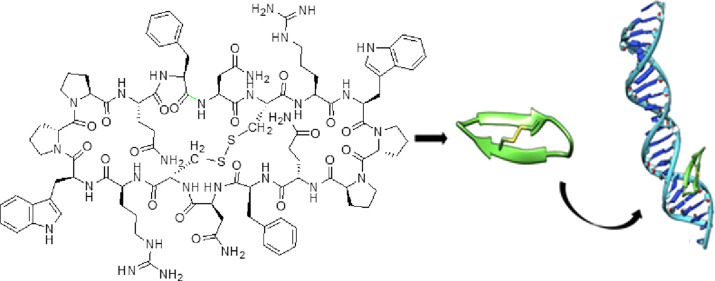

## Introduction

Arc is a homodimeric repressor member of the Ribbon–helix–helix (RHH) family of transcription factors (TFs), encoded by phage P22 and involved in the switch from lysogeny to lysis of *Salmonella typhimurium*. TFs regulate gene expression through recognition and interaction with double-stranded (ds)-DNA. The protein domain that most commonly interacts with the DNA operator is α-helix (HTH family), but the crystal structure determination of the MetJ repressor from *E. coli* in 1989 revealed that an antiparallel β-sheet domain was positioned in the major groove of DNA, contacting DNA bases, which suggested the existence of different families of TFs.^1^

Therefore, the development of proteins of the RHH family is a growing field of research, aiming to mimic TFs and to regulate gene expression, as well as to study the role of flexibility in binding specificity.^2^ Arc is a 53-residue protein consisting of two α-helices, followed by a β-strand at the *N*-terminus, able to bind to DNA as a dimer; here, two β-strands interact together, establishing hydrogen bonds and shaping an antiparallel β-sheet, through which the arc repressor interacts specifically with DNA bases (PDB ID: 1ARR, 1ARQ).

Furthermore, the arc repressor undergoes oligomerization and structures like a dimer of dimers when binding to DNA, positioning the antiparallel β-sheets of the tetramer into two adjacent major grooves of a 21 base pair operator DNA, the so-called TAGA box.^3,4^

Arc folding is led by the formation of a hydrophobic core composed of several residues, which establish hydrophobic interactions, defining the dimer interface.^5^ Residue Phe^10^ from both β-strands is part of the hydrophobic core, and it was recognized as a key amino acid, albeit it does not directly contact DNA bases. In fact, Phe^10^ seems to play a dual role in the binding affinity and specificity to the DNA operator, since in the free protein, the phenyl ring is buried into the hydrophobic core and it flips out when bound to the DNA operator, packing against the sugar–phosphate backbone and establishing additional interactions with DNA.^6^

Moreover, this conformational variation of Phe^10^ allows the correct rearrangement of the key residues toward the DNA bases, in order to establish contact with the TAGA box side chains of Gln^9^, Asn^11^, and Arg^13^ from both β-strands, forming a thick network of hydrogen bonds with six adjacent base pairs of the DNA operator.^3,7^

In recent years, diverse attempts have been made to obtain arc mimetics with topological selectivity for the major groove; both linear and cyclic structures have been prepared by the classic solid-phase peptide synthesis (SPPS) protocol with satisfactory yields and excellent purity, but none of the cyclic ones seem to assume a well-defined 3D conformation.^8,9^

Additionally, the European Union has updated Annex XVII to Regulation (EC) No 1907/2006, including the restriction for the industrial use of *N,N*-dimethylformamide (DMF) and its mixtures; DMF is a preferred solvent for both SPPS and liquid-phase peptide synthesis (LPPS), and its limitation implies the research of solvent alternatives and synthetic procedures.^10^ Even if the literature is full of technical innovations, there are limited reports on the development of green technologies empowering SPPS by sustainable methods.^11^

One of these involves the application of low-frequency ultrasound (US) to SPPS, which paves the way for more green applications. US is an eco-environmental technology based on cavitation, showing diverse advantageous over the canonical heating processes, due to (i) an improvement of the reaction rate, product quality, and yields; (ii) reduction of reaction time; (iii) limited energy consumption and waste production; (iv) use of nonclassical solvents or the absence of solvent use; and (v) use of milder conditions in homogeneous and heterogeneous reactions.^12^

Although US has been already documented as a green approach in diverse reaction systems, its sustainable impact on SPPS by the employment of nontoxic chemicals for the synthesis of arc mimetics, to date, remains to be fully unveiled.

Since the rearrangement into a β-sheet structure is a key feature of a DNA binding-arc mimetic, we envisaged the possibility of building a bicyclic peptide via a green SPPS route for the development of more sustainable processes among the synthetic protocols applied to global constrained peptides.

A complete structural characterization has been performed on the isolated chemical entity after RP-HPLC purification and tandem LC-MS, in order to elucidate the 3D conformational rearrangement in solution. DNA-binding assays have been also performed in order to explore the potential topological selectivity for the DNA major groove.

## Results and Discussion

### Design of the Bicyclic Arc Mimetic

The design of the novel cyclic peptide is based on the incorporation of 16 amino acids into a bicyclic structure containing β-turn inducers, aiming to force the 180° folding of the peptide to a β-hairpin conformation (Figure 1).

**Figure 1:**
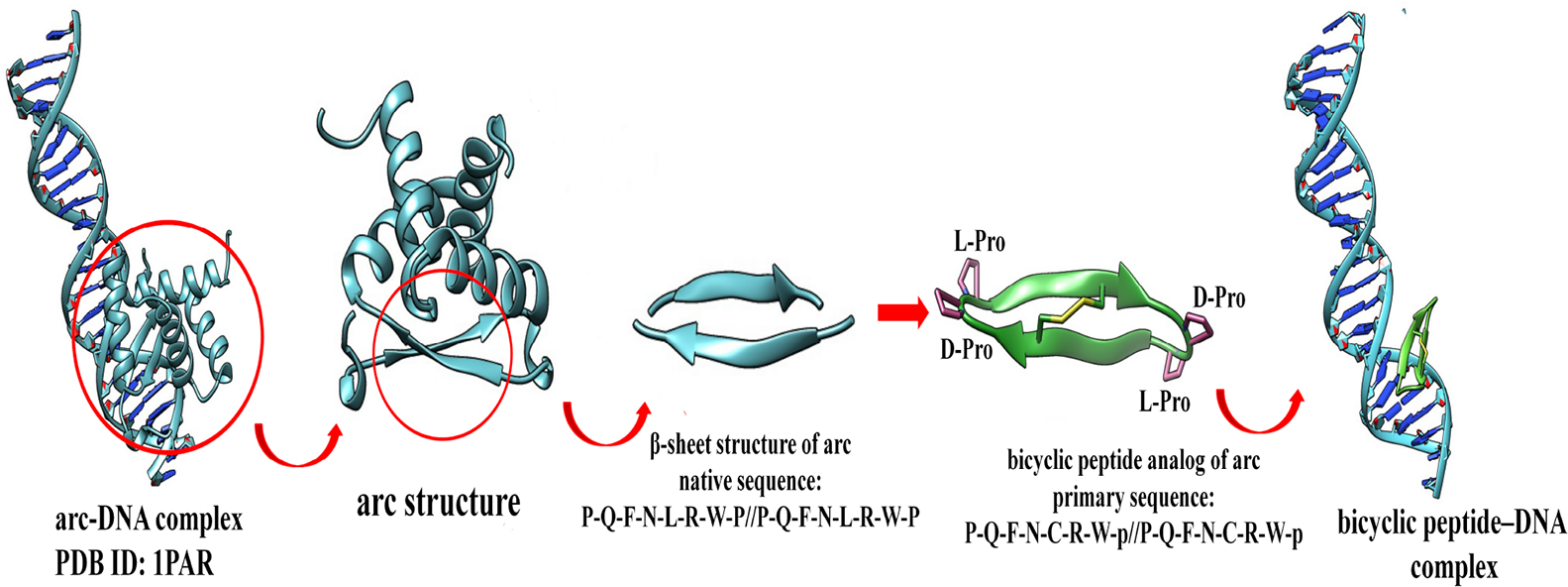
Graphical representation of the design of the bicyclic peptide with Maestro 2021 (Schrödinger, Release 2021–1. Maestro, Schrödinger, LLC, New York, NY, USA; see ref 13).

Starting from the sequence of the β-sheet domain of the native arc repressor #PQFNLRWP//PQFNLRWP#, we chose the d-Pro-l-Pro template as a type II′ β-turn inducer, among the ones previously investigated by us, since its incorporation gave the best percentage of β-sheet secondary structure from the circular dichroism (CD) spectra (primary sequence: Q-F-N-L-R-p-P-Q–F-N-L-R-NH_2_).^9^

A β-turn is stabilized by an intramolecular H-bond and it is able to fold a structure into a β-hairpin; this means that the correct positioning of the d-Pro-Pro nucleating turn (p^16^-P^1^/p^8^-P^9^) inside the macrocyclic structure is fundamental to maintain the paring of side chains through the formation of intramolecular hydrogen bonds promoting the rearrangement into a β-sheet conformation. Even if this structural feature could help the formation of the *head-to-tail* amide bond through the global macrolactamization (Scheme 1, structure 1), it could not be enough to fix the correct 3D conformation of such a peptide. Thus, we applied another local constraint to rigidify the overall structure. Considering that the formation of a monocyclic peptide via I_2_-mediated oxidation of cysteine side chains (Scheme 1, structure 2) allowed the formation of mostly random and α-helix structures (e.g.*,* peptide **9**, primary sequence: Q-F-N-*c*[CR-N-G-QC]­N-L-R-NH_2_; peptide **10**, primary sequence: Q-*c*[CNLR-N-G-QFNC]­R-NH_2_; their 3D structures are displayed in Figure S2; see the SI),^9^ leucine residues were replaced by two cysteine amino acids fully protected at the side chains (Scheme 1, structure 3). The aim is to force the blocking of the 3D conformation via a central disulfide bridge, thus turning the already macrocyclized peptide in a fixed bicyclic structure.

**Scheme 1:**
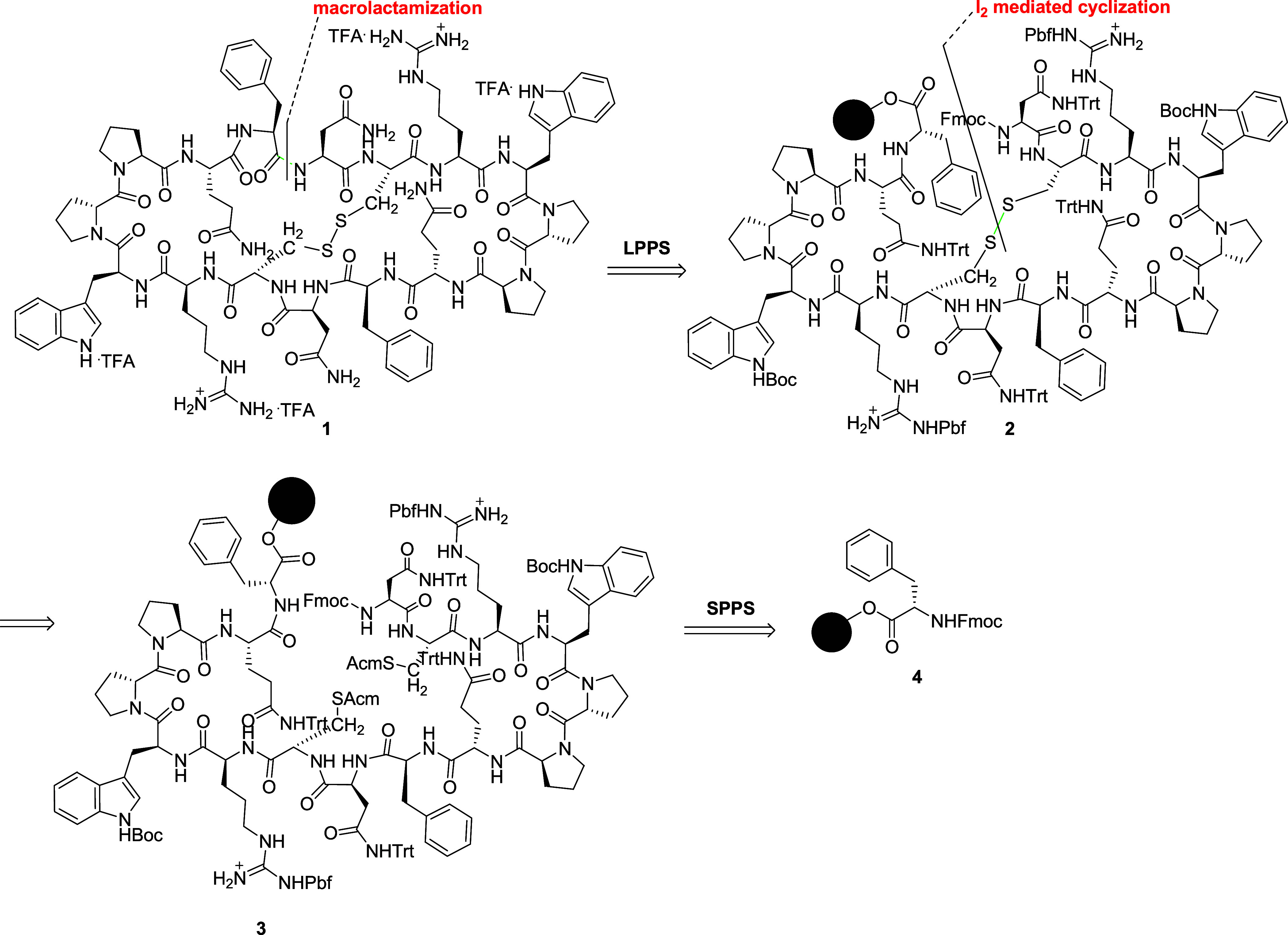
Retrosynthetic Analysis of Newly Designed Bicyclic Peptide 1

### Green Protocol for US-SPPS of the Bicyclic Arc Mimetic

The retrosynthetic plan for the preparation of the title final product 1 is graphically represented in Scheme 1.

The elongation of the primary sequence was performed on a 2-CTC-Cl resin by anchoring Fmoc-Phe-OH (**4**) via the SPPS protocol already described by us with some modifications.^8^

The presence of the two cysteines^5,13^ in the intermediate **3** suggests the possibility to perform the first intramolecular cyclization reaction via the oxidation of *S*-acetamidomethyl (Acm) groups at the side chains so as to obtain intermediate **2**; since the primary sequence of this peptide did not allow the anchoring of COOH-terminal side-chain amino acids, soft cleavage of the fully protected peptide is mandatory before the *head-to-tail* macrocyclization in solution. Finally, full deprotection of the crude peptide gave the desired product **1** as the trifluoroacetic acid (TFA) salt.

In order to comply with the European Commission for Chemicals strategy, “The EU’s chemicals strategy for sustainability toward a toxic-free environment”, we searched for green and easy-to-handle protocols to gain an efficient building of cyclic peptides on resin.

We came across a recent report by Wilson et al. in which they evaluated the use of Cyrene in peptide synthesis through a HATU/DIPEA-mediated coupling protocol in solution.^14^

Results highlight that vigorous stirring appears to be necessary due to the high viscosity of this solvent, even though the stirring rate may be reduced by increasing the stoichiometry of DIPEA, with no negative influence on yields. Furthermore, high yields have been reported for the coupling of several amino acids, also with protected side chains, with the only requirement being to increase the equivalents of HATU in some specific cases. We bypassed these drawbacks by performing a US-SPPS in order to reduce the reaction time for each coupling and to avoid the modification of stoichiometric equivalents for base and coupling reagents. To completely remove Cyrene at the end, basically a series of aqueous washes and trituration with cold diethyl ether were found to be adequate (see the Experimental Section for details).

We performed the coupling reactions for peptide elongation as well as the intramolecular on-resin cyclization using an ultrasonic bath to accelerate the reaction time, HATU, DIPEA, and Cyrene according to the procedure reported in the Experimental Section. Typically, conventional peptide synthesis protocols use a massive amount of solvents, and the most employed ones appear to be DMF and *N*-methyl-2-pyrrolidone (NMP). In particular, DMF is commonly used in SPPS when forming amide bonds, due to its ability to dissolve protected amino acids and promote the swelling of the resins, coupling, and deprotection reactions as well as its suitability for the washing steps.^15^ However, DMF is a highly reprotoxic solvent and is poorly aligned with Green Chemistry guidelines. In this sense, although multiple attempts were carried out to identify suitable solvents alternative to DMF from sustainable sources, it is still a challenging issue, due to the high solubilizing power of this traditional solvent in peptide synthesis.^14^

In 2014, Cyrene, a solvent achievable from cellulose, was used for the first time in organic synthesis in light of its physical properties very similar to those of conventional DMF.^16^

This solvent has been elected as a valuable green alternative due to its high level of safety and nontoxicity to the environment, according to the Global Harmonized System of Classification and Labeling of Chemicals (GHS), since it is readily converted to water and carbon dioxide.^17^

Generally, the solubility of iodine in Cyrene is poor, although it is not considered a suitable solvent for synthesizing iodine-based compounds; this means that it could not be used for the I_2_-mediated oxidation of the elongated peptide on resin. Both cysteines^5,13^ bring acetamidomethyl (Acm)-protecting groups on thiols, which can be orthogonally cleaved, allowing simultaneous disulfide formation.^18^ At this scope, a mixture of I_2_ (5 equiv) in acetonitrile (4 mL) was used under stirring at r.t. for 60 min according to Galanis et al.^19^ in order to facilitate the following structural rearrangement in solution. The so-obtained crude cyclic peptide was then removed from the resin and macrocyclized via LPPS, using HATU (3 equiv) and DIPEA (4 equiv) in Cyrene at r.t. overnight. The crude bicyclic peptide was precipitated with cold ether, washed with water, triturated with cold ether, dried in high vacuum, and lyophilized. The desired peptide was identified by UPLC tandem MS, following purification by RP-HPLC (see SI). The final purity was assessed by analytical RP-HPLC, which was found to be >95%; the overall yield of the bicyclic peptide was around 11.6%.

### CD Spectra of the Bicyclic Peptide

Given the presence of two β-turn inducers and two global constraints in the macrocyclic structure, we first measured the CD spectra of a 50 μM solution of the bicyclic peptide in PBS 1× buffer. The resulting CD spectrum of the peptide shows the typical negative band centered at ca. 210 nm for a β-hairpin structure (Figure 2A).^20^

**Figure 2:**
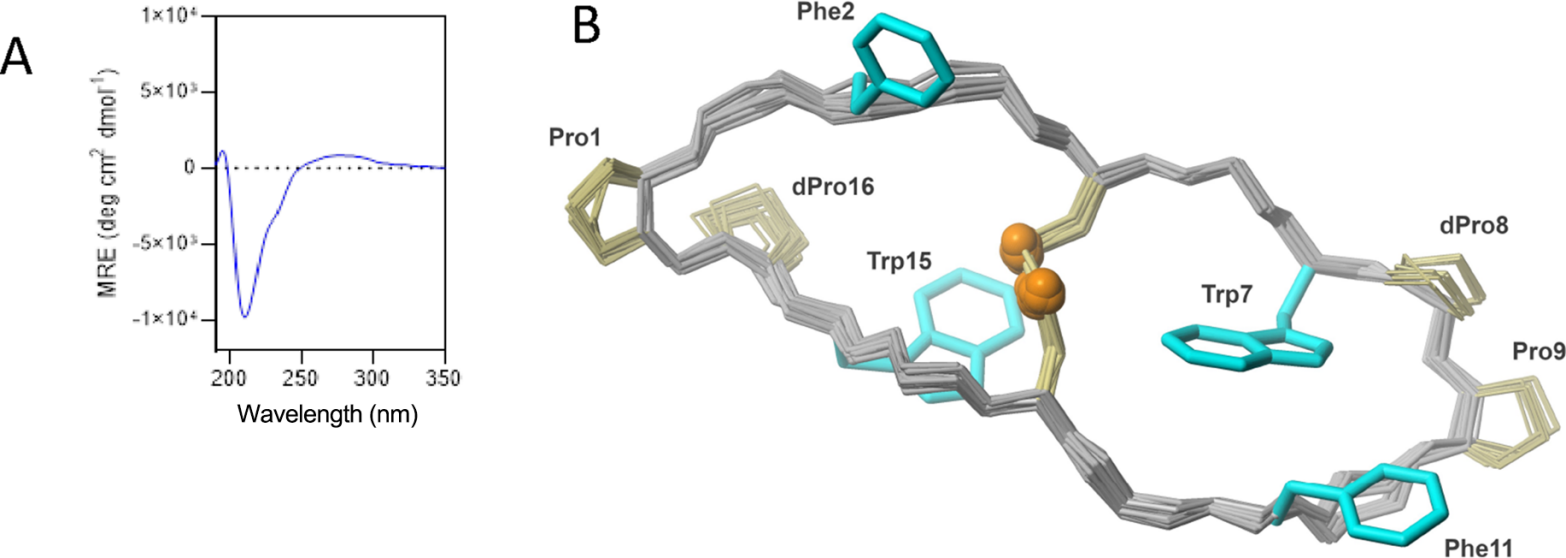
(A) CD spectrum of a solution of a 50 μM final bicyclic peptide in PBS 1× buffer (10 mM, NaCl 100 mM, pH 7.5) at 20 °C. (B) 3D structure of the bicyclic peptide in solution evaluated by NMR spectroscopy. Backbone, d-Pro-Pro motif, and disulfide bond are presented for the 20 refined structures. The side chains for d-Pro-Pro and disulfide are shown in khaki; the side chains for Phe^2,11^ and Trp^7,15^ are highlighted in cyan.

It should be noted that the CD spectrum of a β-hairpin containing D-amino acids is not the same as that of the corresponding all-L peptide. In the case of enantiomeric peptides (all-L versus all-D), the CD spectra are mirror images, reflecting the opposite global chirality (Figure 2B).^21^

However, in mixed-chirality β-hairpins where only loop residues are D-amino acids, the peptides are not enantiomeric. The substitution of D-residues in turns (e*.*g., d-Pro-Gly motifs) has been shown to alter the backbone geometry and β-hairpin stability, leading to measurable differences in the CD spectrum.^22^

### NMR Structural Analysis

The NMR data were acquired for the bicyclic peptide obtained on an Agilent DDR2 800 NMR spectrometer at 298 K, which enabled the assignment of nearly 90% of the ^1^H, ^13^C, and ^15^N resonances presented in the peptide (Table S1, see the SI). The 3D structure was evaluated based on 65 distance constraints derived from the ^1^H-^1^H NOESY spectrum. Additionally, there are 30 restraints on the φ and ψ backbone torsion angles and four constraints for two hydrogen bonds derived from the NMR data (Table S2, see the SI). The 3D structure of the peptide in solution is described as two (six residues long) fragments, comprised of ^2^QFNCRW^7^ and ^10^QFNCRW^15^. Residues in those fragments exist in an extended conformation close to a β-sheet. They are connected with the d-Pro-l-Pro motifs, which form β-turns on both sides of the peptide. The central disulfide bond between Cys^5^ and Cys^13^ provides an additional stabilization effect. The performed calculations resulted in a well-defined 3D structure, with an rmsd of 0.41 ± 0.15 Å for the 20 refined conformers (Table S2; see the SI).

### Tryptophan Fluorescence Assay

Once we confirmed the β-hairpin structure for the peptide, we analyzed its binding to two different DNA sequences. The DNA-binding properties of the bicyclic peptide were investigated by using fluorescence spectroscopy. Given the presence of two tryptophan residues in its sequence, intrinsic tryptophan fluorescence was employed to monitor its interaction with DNA. Tryptophan fluorescence is highly sensitive to its local environment, and fluorescence quenching has been widely used to probe conformational changes in proteins as well as protein–DNA interactions.^23,24^

In the context of DNA binding, quenching generally reflects the close proximity between indole side chains and DNA bases, often due to electron transfer, but it does not by itself establish a specific binding mode. As anticipated, the addition of the TAGA hDNA to a solution of the peptide resulted in a marked decrease in the tryptophan fluorescence intensity (Figure 3), supporting direct interaction between the peptide and DNA.^24,25^

**Figure 3:**
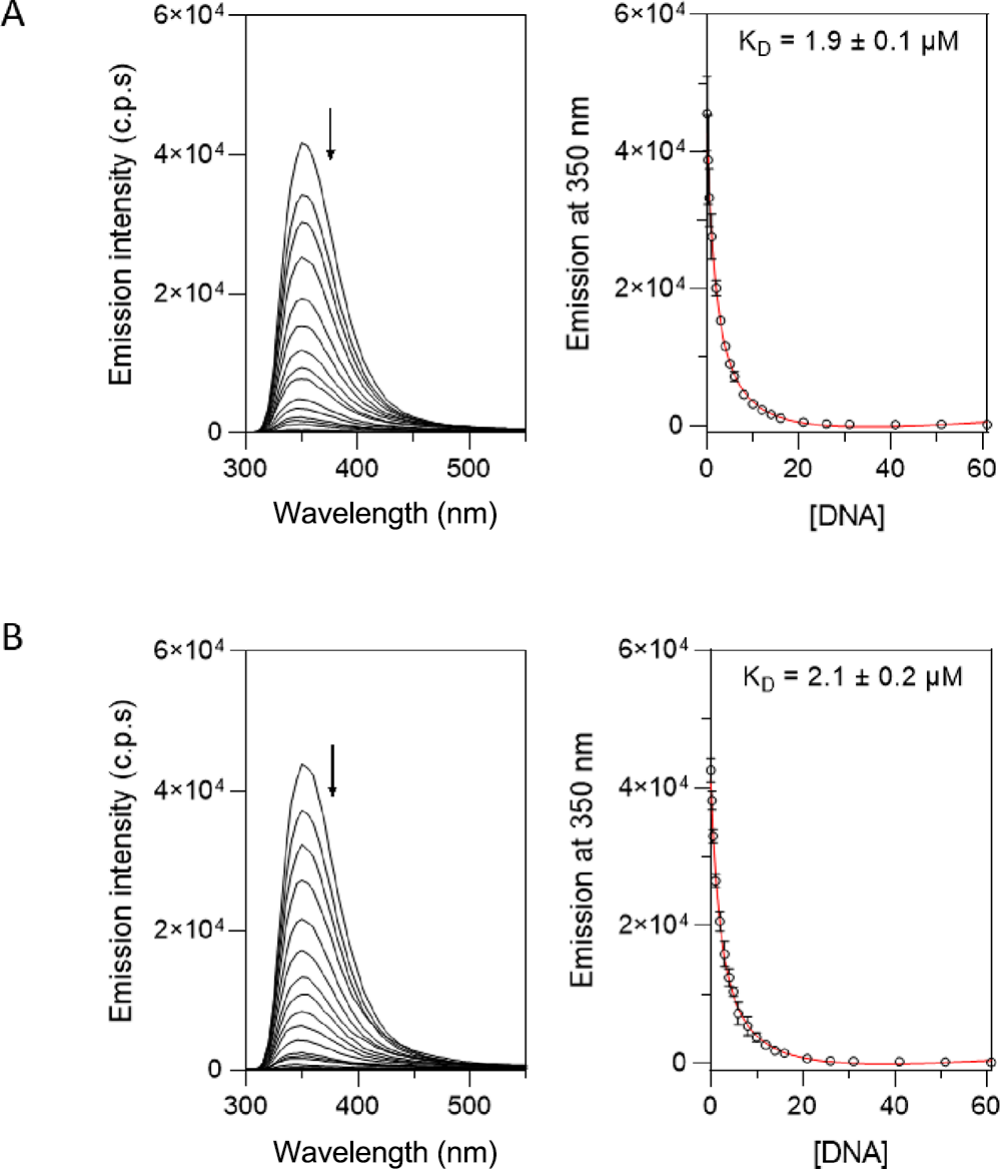
(A) Left: fluorescence spectra of a 0.5 μM solution of the peptide with TAGA hDNA. Right: titration profile of the maximum emission wavelength at 350 nm with increasing concentrations of DNA, showing the best fit to a 1:1 model. Experimental data points correspond to the average of three independent titrations. (B) Left: fluorescence spectra of a solution 0.5 μM of peptide with increasing concentrations of CACA hDNA. Right: titration profile of the maximum emission wavelength at 350 nm with increasing concentrations of DNA. The best fit according to the 1:1 model in *Dynafit* is also shown. Experimental data points correspond to the average of three independent titrations.

The DNA binding constants were numerically calculated using *Dynafit* software (Biokin, Ltd.).^26,27^ We obtained a dissociation constant (*K*_D_) of 1.9 μM for the binding of bicyclic peptide with target TAGA hDNA, which is a constant quite similar to that in a previously published work using a similar cyclic peptide.^8^ Interestingly, the peptide displayed a binding behavior nearly identical to those of both TAGA hDNA and the nonconsensus sequence CACA hDNA, with a *K*_D_ of 2.1 μM (Figure 3).

### Thermal Denaturation Experiments

To gain further insights into the DNA-binding properties of the peptide, we carried out thermal denaturation experiments by monitoring changes in the CD spectrum of the DNA.^28–30^

Initially, 10 μM solutions of both DNA sequences were analyzed in the absence of the peptide by gradually increasing the temperature from 15 to 97 °C at a rate of 2 °C per minute. CD signals were recorded at 250 nm (Figure 4). For TAGA hDNA, a melting temperature (*T*_m_) of 76 °C was observed, while CACA hDNA exhibited a slightly higher *T*_m_ of 81 °C.

**Figure 4:**
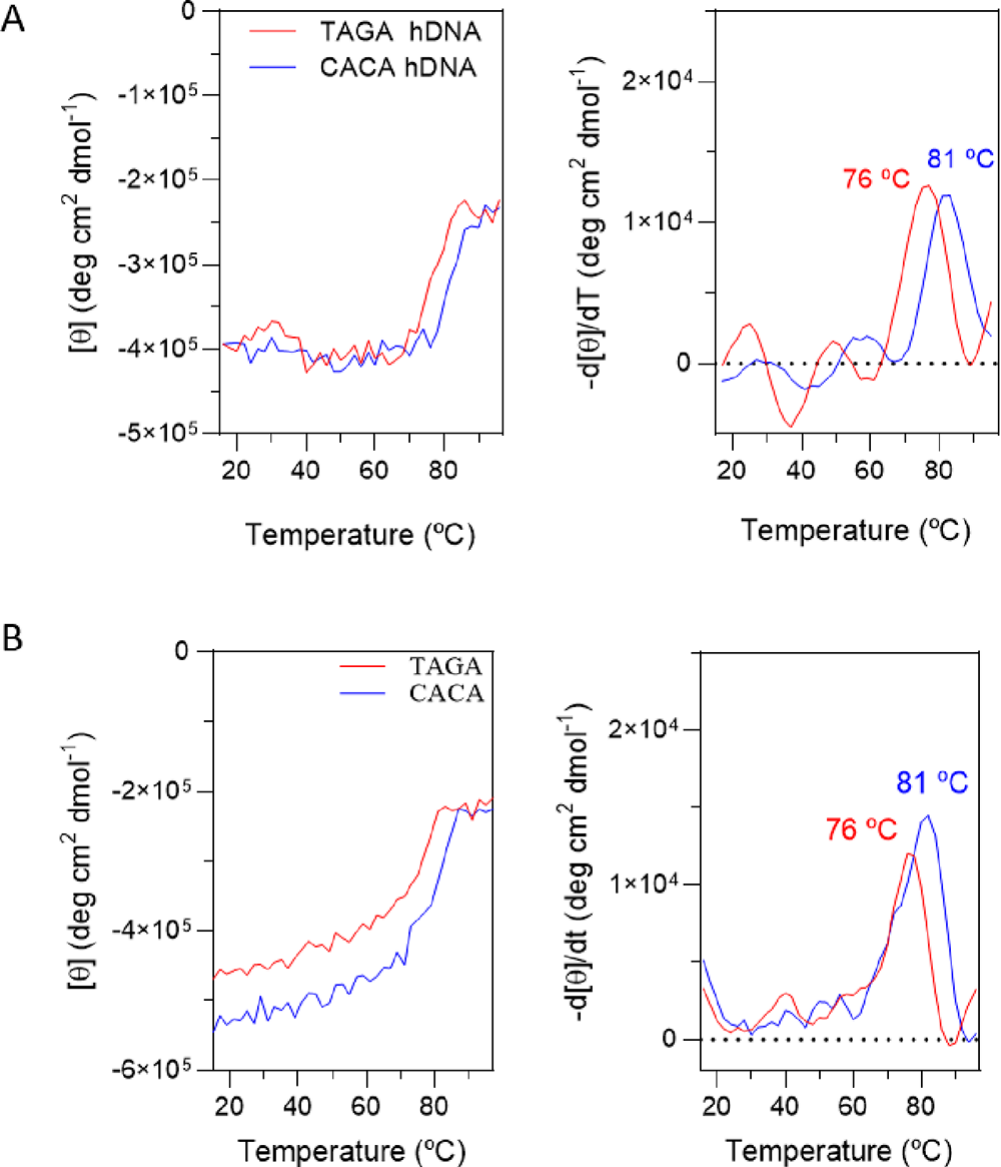
(A) Left: thermal denaturation profile of TAGA (red line) and CACA (blue line) hDNAs. Right: first-order derivative of the profiles. The experiment was carried out in PBS 1× buffer (10 mM, NaCl 100 mM, pH 7.5) from 15 to 97 °C at a ratio of 2 °C/min. (B) Left: thermal denaturation profile of DNA–peptide complexes. Right: first-order derivative of the profile. The experiment was carried out in PBS 1X buffer (10 mM, NaCl 100 mM, pH 7.5) from 15 to 97 °C at a ratio of 2 °C/min.

Subsequently, the same thermal denaturation protocol was applied to peptide–DNA complexes using a 1:1 DNA-to-peptide molar *ratio* (10 μM each). The thermal denaturation values remained unchanged upon peptide binding: 76 °C for the TAGA hDNA–peptide complex and 81 °C for the CACA hDNA–peptide complex, indicating that the peptide does not significantly alter the thermal stability of either DNA duplex (Figure 4).

### Docking Experiments

Finally we performed *in silico* docking with the aim of elucidating the possible interactions that the bicyclic mimetic could establish with TAGA and CACA box sequences. Experiments were performed according to the methods described in the Experimental Section. When docked to TAGA DNA, the bicyclic peptide exhibited a docking score of −6.3 kcal/mol; however, when docked to CACA DNA, it showed a docking score of −6.5 kcal/mol, with a similar pose in both docking experiments (Figure S3). The interactions established by the native β-sheet domain of arc repressor with the TAGA box inside the crystal structure have been investigated using UCSF Chimera 1.16.^31^ The arc repressor establishes a thick net of interactions through the β-sheet domain, made up of H-bonds with nucleobases; the residues that specifically interact with DNA are Gln9, Asn11, and Arg13 from the two β-strands. In the crystallographic structure of arc repressor binding to TAGA DNA, Asn 11.B interacts with DT 6, while Gln 9.B, Asn 11.A, and Arg 13.A interact with DA 7, and Arg 13.A interacts also with DG 8 and DA 9. In addition to the specific interactions with the TAGA box, Gln 9.A interacts with DG 5 and DA 18, Asn 11.B establishes H-bonds with DT 17 and DA 18, while Arg 13.B displays interactions with DA 4 and DG 5 (Figure S3A).

Considering the interactions established by the native arc repressor, we analyzed the contacts that the novel peptide forms with TAGA DNA and CACA DNA (see Table S3). When docked to TAGA DNA, the peptide displays H-bonds with DT 6, DA 7, DG 8, DA 9, and DT 15 nucleobases, along with H-bonds with DA 4 and DG 5 nucleobases. Moreover, Gln 9.A, Asn 11.A, and Trp 14.A form ionic interactions with the phosphate group of DA 4, DG 5, and DA 7 nucleotides (Figure S3B).

On the other hand, the docking of peptide to CACA DNA gave a docking score of −6.5 kcal/mol for the best pose. In this case, the peptide interacts with DA 7 and the mutated DC 8 nucleobases through H-bonds, but Gln 9.A, Arg 13.A, and Arg 13.B form ionic interactions with the phosphate group of DA 4 and DT 15, which is approximately 50% of the contacts (Figure S3C). Ionic interactions are stronger and less specific contacts compared to H-bonds established with nucleobases. This point may explain the similar binding of the peptide to TAGA and CACA DNA oligomers, supported also by the similar docking score values obtained. With the latter, the novel peptide establishes fewer interactions overall; however, nearly 50% are ionic interactions with phosphate groups, which nonspecifically enhance the binding of the peptide to the control sequence.

## Conclusions

In this study, we report the synthesis of a β-sheet-like bicyclic peptide designed as a mimetic of the arc repressor, with the aim to develop a biomimetic scaffold capable of recognizing specific DNA binding sequences. The peptide was synthesized using a green SPPS approach, contributing to the advancement of more sustainable methodologies for the preparation of globally constrained peptides. The β-sheet conformation of the resulting peptide was confirmed by NMR and CD spectroscopies. Binding studies revealed that it exhibits a strong affinity for hDNA, although with limited sequence selectivity. Further studies are necessary to improve the specific sequence recognition of the DNA to better delineate the functional features of this novel β-sheet arc mimetic.

## Experimental Section

### Chemicals

HPLC-grade solvents were acquired from Merck (Milano, IT). 2-Chlorotrityl chloride resin, HATU, DIPEA, iodine, Cyrene, methanol, dichloromethane, and all Fmoc-protected amino acids were purchased from Novabiochem (Massachusetts, USA) and Merck (Milano, IT). Biochemical-grade TFA for HPLC was acquired from Acros Organics (Segrate (MI), IT), and standard-grade TFA for the deprotection of peptides was purchased from Fischer Scientific (Pittsburgh, PA). D_2_O and DMSO-*d*_6_ were acquired from EurisoTop (Saint Aubin Cedex, France); all other reagents were from Aldrich Chemical Co.

### Peptide Synthesis

The elongation and I_2_-mediated oxidation of the linear peptide were carried out on 2-CTC-Cl resin (1.60 mmol/g) by US-SPPS synthesis on an ultrasonic bath, unless specified.

*SONOREX* RK 52 H as an ultrasonic bath with interior dimensions of 150 × 140 × 100 mm, operating volume 1.2 L, by BANDELIN electronic (Germany), was equipped with a timer control (1–15 min), continuous (∞) operations, and built-in heating control (30–80 °C), an ultrasonic frequency of 35 kHz, an ultrasonic nominal output of 60 W, an ultrasonic peak output of 240 W corresponding to 4 times the ultrasonic nominal output, and a heating power of 140 W. The 2-CTC-Cl resin (300 mg, 1.6 mmol/g, 0.48 mmol) was poured into a SPPS plastic syringe (Torviq syringe, Tucson Arizona, USA) and swelled in 3 mL of dry CH_2_Cl_2_ for 40 min. Then, a mixture of Fmoc-Phe-OH (3 equiv, 557.9 mg, 1.44 mmol) in 4 mL of CH_2_Cl_2_ and DIPEA (6 equiv, 372.2 mg, 0.5 mL) was added to the resin and shaken for 6 h. The resin was drained, washed 6 times with CH_2_Cl_2_ (5 mL), and capped 2 times with 17:2:1 CH_2_Cl_2_/CH_3_OH/DIPEA (5 min each). After washing with dichloromethane (3 times, 5 mL each), the resin was subjected to cycles of US-SPPS using amino acid building blocks: Fmoc-Trp­(Boc)–OH, Fmoc-Arg­(Pbf)–OH, Fmoc-Cys­(Acm)–OH, Fmoc-Asn­(Trt)–OH, Fmoc-Phe-OH, Fmoc-Gln­(Trt)–OH, Fmoc-d-Pro-OH, and Fmoc-Pro-OH. Couplings and Fmoc-deprotection reactions were performed in the ultrasonic bath following the procedures previously described by Stefanucci et al.^32^

After each coupling reaction, a Kaiser/chloranil test was performed to check the completeness, and then, the *N*-terminal Fmoc group was removed with piperidine 20% in DMF (5 mL, 2 times, 15 min each), and the resin was washed with DMF and dichloromethane. For the coupling reaction, the new protected amino acid (3 equiv, 1.44 mmol) was activated by HATU (3 equiv, 1.44 mmol) and DIPEA (4 equiv, 1.92 mmol, 0.34 mL) in Cyrene (4 mL); each coupling reaction proceeds in 15 min and each deprotection is completed in 30 min.

### Disulfide Bridge Formation

Before cleavage from the solid support, I_2_ oxidation of the thiol groups was performed with a mixture of I_2_/acetonitrile (5 equiv/4 mL) in an ultrasonic bath at 25 °C for 1 h. Then, the resin was washed with CH_2_Cl_2_ (3 times) and DMF (3 times).

### Cleavage of Peptide

The resin was carefully washed with DMF (5 mL) once and with CH_2_Cl_2_ (5 mL) 3 times, and a mixture of 1% TFA/CH_2_Cl_2_ (5 mL) was added to the syringe under shaking at r.t. for 1 h; then, the solution was collected in a 250 mL flask, and the resin was treated with CH_2_Cl_2_ (5 mL) 4 times; the solution was concentrated until 1 mL on a rotary evaporator and precipitated in cold ether. The crude solid was washed and centrifuged with cold ether 5 times, and then it was dried under high vacuum to obtain a crude product as a white solid in approximately 24% (0.236 g) unpurified yield. *Cyclization.* HATU (3 equiv, 0.039 mmol, 14.83 mg) and DIPEA (4 equiv, 0.052 mmol, 9.06 μL) were dissolved in Cyrene (50 mL) in a 250 mL round-bottom flask; 50 mg of the crude peptide was dissolved in 20 mL of Cyrene and slowly added to the reaction mixture over 1 h under stirring. The solution was then stirred for 24 h at r.t.; the crude peptide was precipitated with distilled water, and the solid residue triturated with water 5 times. The crude product was dried in a rotary evaporator under high vacuum for 3 h. Around 1 mg of crude fully protected peptide was dissolved in 1 mL of CH_3_OH for RP-UPLC analytical trace (C18-bonded 4.6 mm × 150 mm), using the following parameters: flow rate 1 mL min^–1^; linear gradient of water/ACN 0.1% TFA starting from 10% ACN to 90% ACN in 20 min (λ 214 nm). The peak corresponding to the desired intermediate shows an rt of 5.03 min (*m*/*z*: 3729.9 [M]). Then, the crude bicyclic peptide was directly deprotected without purification.

### Deprotection and Purification

The protected bicyclic peptide (86 mg) was dissolved in 10 mL of 18:1:1 TFA/TIPS/H_2_O in a 25 mL round-bottom flask at rt under stirring for 1 h; then, 5 mL of water was poured into the solution and kept under stirring for another 15 min; then, the solution was concentrated to an oil in a rotary evaporator and precipitated into 2 vials containing cold ether (3 mL each). The supernatant was removed, and the solid residue was washed 5 times with cold ether after 5 steps of centrifugation (4400 rpm, 60 s each). The residue was dissolved in 2 mL of water, placed in a lyophilizator for 48 h, and analyzed by analytical RP-UPLC as described above (*R_t_* 4.32 min, *m*/*z*: 2056.5 [M + H]^+^). After purification on a semi-Prep RP-HPLC (gradient of 5–90% ACN in 32 min), the purity of the final compound as the TFA salt was determined to be ≥95%.

The crude final peptide was purified by RP-HPLC using a Luna Phenomenex semi-Prep C18, 5.0 μm, 250 × 10 mm column, at a flow rate of 5 mL/min on a Waters pump 600, using as the eluent a linear gradient of H_2_O + 0.1% TFA/ACN + 0.1% TFA, from 5% ACN to 90% ACN in 32 min.

Lyophilization of the pure fractions yielded the peptide as a white fluffy solid in 3% yield (0.021 g).

### HPLC and Mass Spectra

For analytical RP-HPLC was used a Water system equipped with a 660 pump and C18 analytical RP-HPLC column (XBridge Waters column C18, 130 Å, 3.5 μm 4.6 × 150 mm) at 216, 235, 254, and 275 nm, at a flow rate of 1 mL/min, using as eluent a gradient of H_2_O/ACN-0.1% TFA ranging from 5% ACN to 90% ACN over 30 min. LRMS was performed on a LCQ Finnigan-Mat mass spectrometer (San Jose, CA) by an ESI-spray source and an ion trap analyzer, with a capillary temperature of 200 °C and a spray voltage of 4.00 kV. Nitrogen (N_2_) and helium were used as both the sheath gas and the auxiliary gas.

### NMR Spectroscopy

The NMR sample was obtained by dissolving 1.5 mg of the bicyclic peptide in H_2_O/D_2_O 90%/10% solvent. Due to its low solubility in water, a small amount of acetic acid-d_4_ was added to increase the concentration of peptide in solution. A Shigemi tube (Rototec-Spintec GmbH, Bad Wildbad, Germany) was used for NMR measurements to decrease the sample volume.

All NMR experiments were performed at 298 K on Varian Inova 500 (^1^H resonance frequency 500.606 MHz) and Agilent DDR2 800 NMR spectrometers (^1^H resonance frequency 799.613 MHz). Both spectrometers were equipped with three channels, a z gradient unit, and a ^1^H/^13^C/^15^N triple-resonance probe head with inverse detection. In some cases, to increase the dispersion of the ^1^H resonances, NMR experiments were performed at 303 K.

The assignments of the ^1^H, ^13^C, and ^15^N resonances were achieved with joined analysis 2D homonuclear and heteronuclear (^1^H-^13^C and ^1^H-^15^N) experiments. The ^1^H-^1^H TOCSY data were recorded with mixing times of 40 and 80 ms using the MLEV-17 pulse scheme for spinlock.^33^ The NOESY experiment was performed with a mixing time of 120 ms. The ^13^C and ^15^N resonances were assigned on base 2D ^1^H-^13^C HSQC and ^1^H-^15^N HSQC detected on the natural abundance of ^13^C and ^15^N isotopes. All chemical shifts were referenced with respect to external sodium 2,2-dimethyl-2-silapentane-5-sulfonate (DSS) using Ξ = 0.251449530 and 0.101329118 ratios for indirectly referenced ^13^C and ^15^N resonances, respectively.^34^ All recorded NMR data were processed by NMRPipe software^35^ and analyzed with the NMRFAM-Sparky program.^36^

### Evaluation of the 3D Structure

Evaluation of the 3D structure calculations of the peptide was performed with CYANA (version 3.98.13) software,^37^ based on 65 unique (30 intraresidue, 21 sequential, 3 medium, and 11 long-range) ^1^H-^1^H distance constraints yielded from the analysis of the ^1^H-^1^H NOESY spectrum acquired with a mixing time of 120 ms. The additional 30 restraints to the backbone φ, ψ, and χ_1_ torsion angles were deduced with TALOSn software^38^ from assigned ^1^H, ^13^C, and ^15^N resonances. The chemical shifts of the ^13^Cβ nuclei in Cys^4^ and Cys^12^ (43.301 ppm) indicate that the thiol groups are in an oxidized state,^39^ confirming the existence of a disulfide bond in the studied peptide. The topology of d-Pro was initially created with Yasara software and transformed into a cyana library. The conformations of the d-Pro-l-Pro peptide bonds were defined as *trans* based on ^13^Cγ/^13^Cβ chemical shifts^40^ and PROMEGA software.^41^ The final refinement procedure in explicit solution (water box) was performed with Yasara (version 20.12.24) software^37,42^ for both peptides utilizing modified versions of nmr_refine.mcr, and nmr_setdefault.mcr macros included in the Yasara library.

### Circular Dichroism

CD measurements were performed with a Jasco J-1100 CD spectrometer coupled to a Jasco MCB-100 mini circulation bath, using a Hellma 100-QS cuvette (2 mm light pass) dissolving the peptide and DNA in PBS buffer 1×. The scan speed was 200 nm/min, the bandwidth was 2.0 nm, the resolution was 0.5 nm, the accumulation was for 3 scans, and the sensitivity was 20 mdeg. Thermal denaturation experiments were carried out using 10 μM solutions of the peptide:DNA complex (1:1 ratio) from 15 to 95 °C at a ratio of 2 °C/min.

### Fluorescence Spectroscopy

Luminescence experiments were carried out in a Jobin-Yvon Fluoromax-3 (DataMax 2.20) coupled to a temperature controller, Wavelength Electronics LFI-3751. All measurements were performed with a Hellma semimicro cuvette (108F-QS) at 20 °C. The tryptophan emission spectra of the peptide (0.5 μM) were recorded in PBS 1× buffer (10 mM, NaCl 100 mM, pH 7.5). Settings: increment, 1.0 nm; integration time, 0.1 s; excitation slit width, 4.0 nm; emission slit width, 6.0 nm at 20 °C.

### Docking Experiments

The DNA structure containing the consensus sequence TAGA, in complex with arc repressor, was retrieved from the RCSB database (PDB ID: 1PAR), and the raw crystallographic structure was prepared using Prep Wizard tool embedded in Maestro Schrödinger 2021–1.^13^

Briefly, the protonation state was estimated by PropKa at pH 7.0, and a minimization was then performed using the OPLS4 force field. To obtain the mutated sequence CACA, the DT6 and DG8 nucleotides were replaced with DC nucleotides and the ds-DNA obtained was reprepared following the same protocol used for TAGA DNA. The bicyclic peptide was docked to TAGA- and CACA-containing DNA by using AutoDock Vina,^43^ obtaining 8 poses for each DNA oligomer. The docking grid was manually centered at the native arc β-sheet. The docking was performed by keeping the ds-DNA rigid and the ligand flexible. We selected the best pose for each DNA oligomer in order to analyze the net interaction. The three-dimensional structures of peptides 9 and 10 were drawn using Maestro Schrödinger 2021 and prepared by LigPrep tool in Maestro, using Epik at pH 7.0 and the OPLS4 force field. The structures were then aligned and superimposed on the bicyclic peptide using UCSF Chimera version 1.16.

## Supplementary Material



## Data Availability

The data underlying this study are available in the published article and its Supporting Information.

## Notes

UPLC-MS and RP-HPLC analytical traces, NMR assignments and experiments, and *in silico* docking experiments (PDF)

## Abbreviations

TF: transcription factor
RHH: ribbon–helix–helix
US-SPPS: ultrasound-assisted solid-phase peptide synthesis
LPPS: liquid-phase peptide synthesis
RP-HPLC: reverse-phase high-performance liquid chromatography
LC-MS: liquid chromatography mass spectrometry
UPLC: ultraperformance liquid chromatography
CTC-Cl: 2-chlorotrityl chloride resin
HATU: O-(7-azabenzotriazol-1-yl)-N,N,N′,N′-tetramethyluronium hexafluorophosphate
DIPEA: N,N-diisopropylethylamine
Pbf: 2,2,4,6,7-pentamethyldihydrobenzofuran-5-sulfonyl group
Acm: acetamidomethyl
Trt: trityl
DMF: N,N-dimethylformamide
NMP: *N*-methyl-2-pyrrolidone
r.t.: room temperature
CD: circular dichroism
NMR: nuclear magnetic resonance
DMSO: dimethyl sulfoxide
ACN: acetonitrile
TFA: trifluoroacetic acid
TIPS: triisopropylsilane
LRMS: low-resolution mass spectrometry
NOESY: nuclear Overhauser enhancement spectroscopy
HSQC: heteronuclear single quantum coherence
DSS: sodium 2,2-dimethyl-2-silapentane-5-sulfonate
PBS: phosphate-buffered saline
