# Correction to “Greening the Solid-Phase Peptide Synthesis of the First Bicyclic Analogue of the Arc Repressor and Its Binding to DNA”

**DOI:** 10.1021/acs.joc.5c03128

**Published:** 2025-12-30

**Authors:** Eleonora Procino, David Bouzada, Sara D’Ingiullo, Lorenza Marinaccio, Igor Zhukov, Azzurra Stefanucci, Adriano Mollica

In a sentence near the end of the Introduction [after citation of ref 12], a reference citation was omitted. It is added here as ref 1:

“Although US has been already documented as a green approach in diverse reaction systems,^1^ its sustainable impact on SPPS by the employment of nontoxic chemicals for the synthesis of arc mimetics, to date, remains to be fully unveiled.”
