# Supplementary material for: Greening the Solid-Phase Peptide Synthesis of the First Bicyclic Analogue of the Arc Repressor and Its Binding to DNA
Source: J Org Chem. 2025 Nov 11;90(46):16423–31. doi: 10.1021/acs.joc.5c01902 (PMC12645479; doi:10.1021/acs.joc.5c01902)
Supplement: Supplementary file 1 [file jo5c01902_si_001.pdf]

SUPPORTING INFORMATION

GREENING THE SOLID PHASE PEPTIDE SYNTHESIS OF THE FIRST BICYCLIC ANALOGUE OF ARC REPRESSOR AND ITS BINDING TO DNA

Eleonora Procino,[1,°] David Bouzada,[2,°] Sara D'Ingiullo,[1] Lorenza Marinaccio,[3] Igor Zhukov,[4] Azzurra Stefanucci,[1,*] Adriano Mollica[1]

[1]Department of Pharmacy, University of Chieti-Pescara "G. d'Annunzio", Via dei Vestini 31, 66100 Chieti, Italy.

[2]Centro Singular de Investigación en Química Biolóxica e Materiais Moleculares (CiQUS), Departamento de Química Orgánica, Universidade de Santiago de Compostela, 15782, Santiago de Compostela, Spain.

[3]Department of Innovative Technologies in Medicine and Dentistry, University "G. d'Annunzio" of Chieti-Pescara, Via dei Vestini, 66100 Chieti, Italy.

[4]Laboratory of Biological NMR, Institute of Biochemistry and Biophysics, Polish Academy of Sciences, ul. Pawińskiego 5A, 02-106 Warsaw, Poland.

*a.stefanucci@unich.it

°these two authors contributed equally.

**TABLE OF CONTENTS**	PAGES
UPLC-MS traces and RP-HPLC purity of final bicyclic peptide	2-6
NMR assignments and spectra for final bicyclic peptide	7-9
In silico experiments	10-13

UPLC-MS traces for final bicyclic peptide C96H126N28O20S2

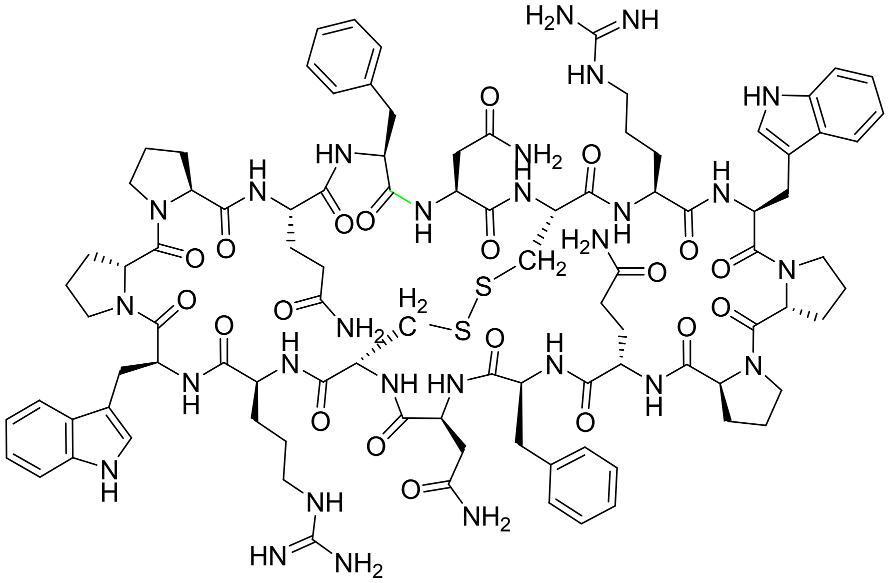

$C_{96}H_{126}N_{28}O_{20}S_2$
Exact Mass: 2054.91
Mol. Wt.: 2056.33
m/e: 2055.92 (100.0%), 2054.91 (94.3%), 2056.92 (58.2%), 2057.92 (28.0%), 2056.91 (19.3%), 2055.91 (11.3%), 2057.91 (10.6%), 2058.92 (7.8%), 2058.93 (6.8%), 2059.92 (2.9%), 2059.93 (1.9%), 2058.91 (1.6%), 2057.93 (1.1%)
C, 56.07; H, 6.18; N, 19.07; O, 15.56; S, 3.12

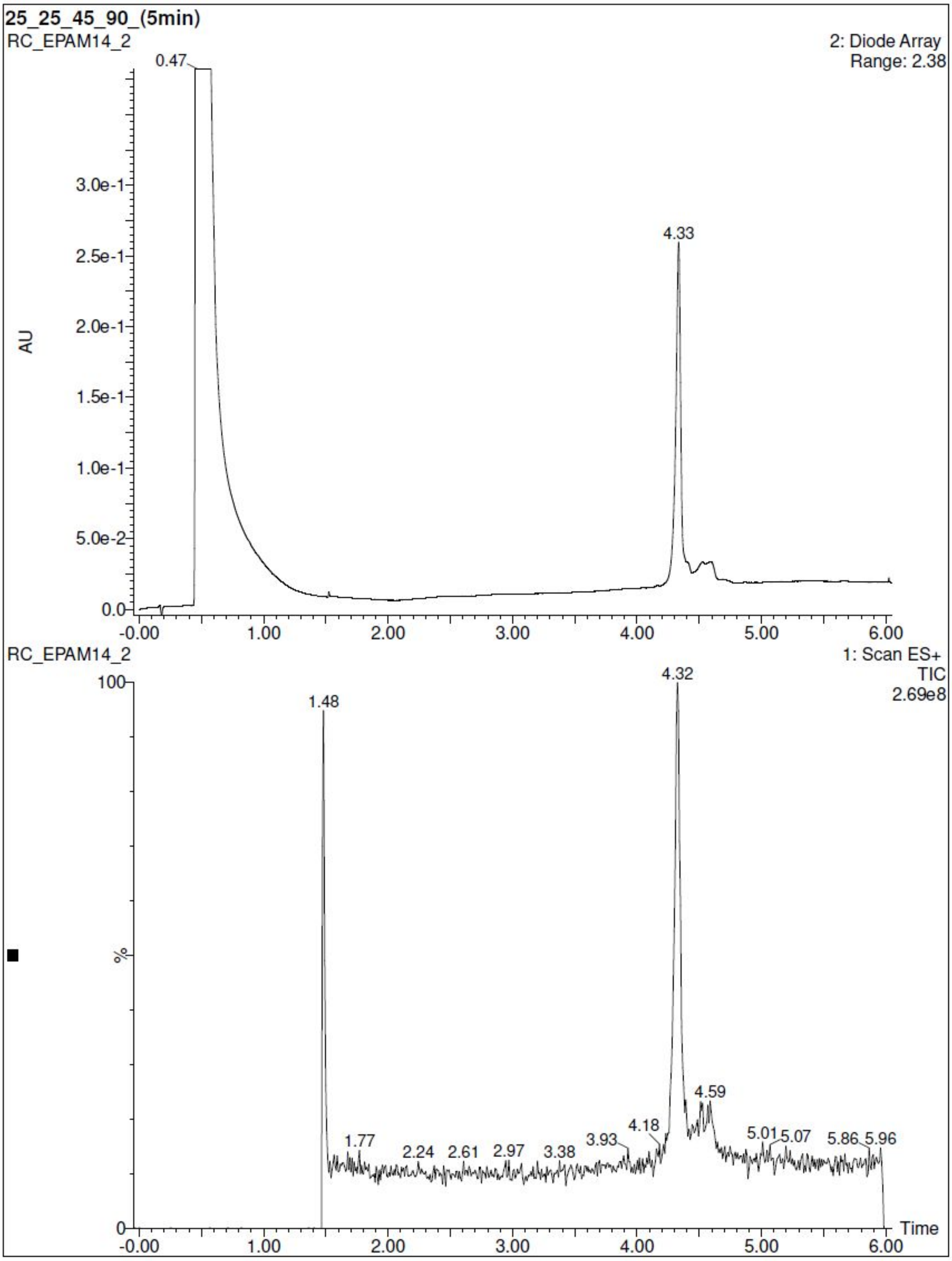
25_25_45_90_(5min)
RC_EPAM14_2
2: Diode Array
Range: 2.38
0.47
4.33
AU
3.0e-1
2.5e-1
2.0e-1
1.5e-1
1.0e-1
5.0e-2
0.0
-0.00
1.00
2.00
3.00
4.00
5.00
6.00
RC_EPAM14_2
1: Scan ES+
TIC
2.69e8
100
1.48
4.32
%
1.77
2.24
2.61
2.97
3.38
3.93
4.18
4.59
5.01
5.07
5.86
5.96
0
Time

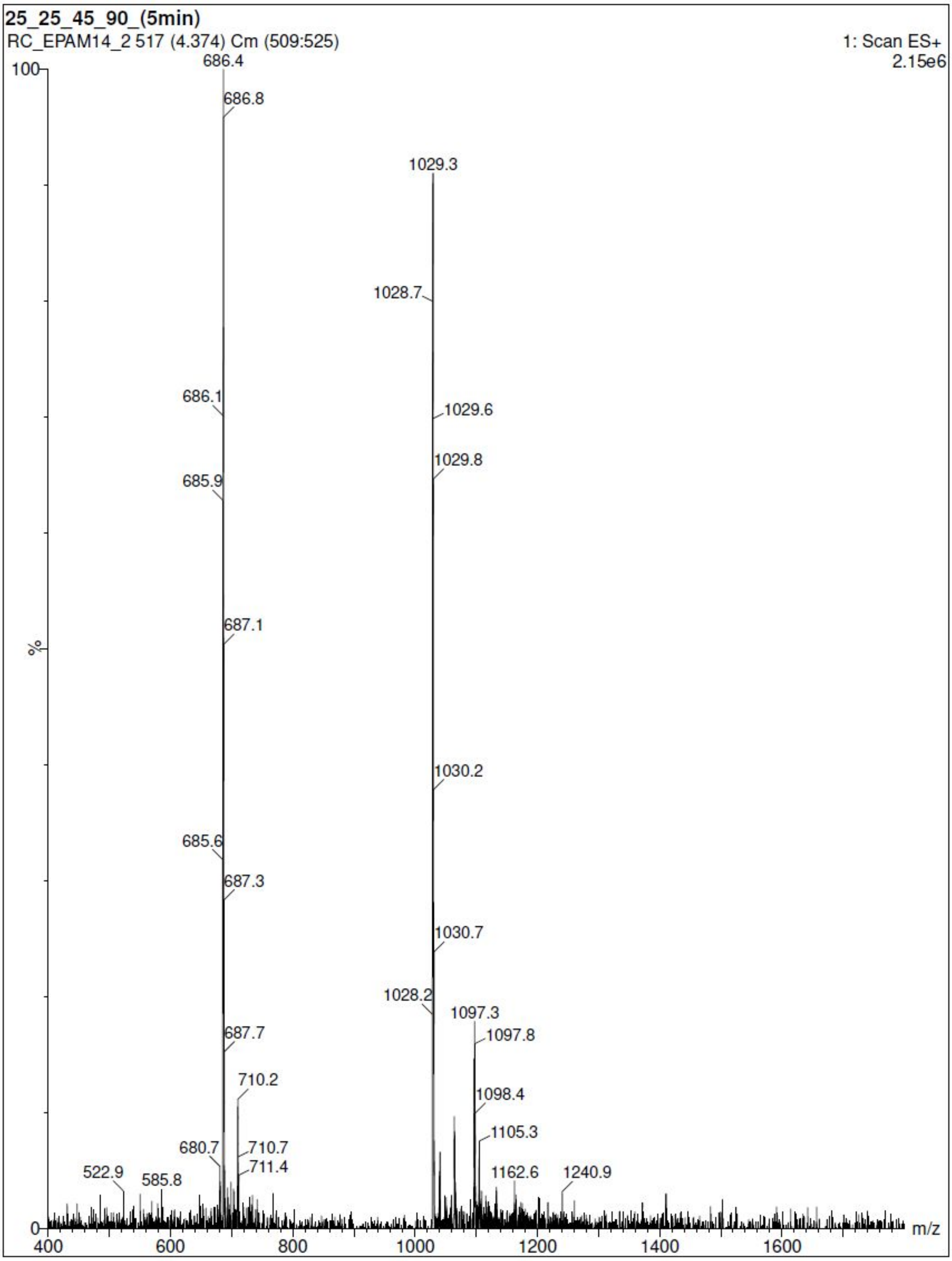
25_25_45_90_(5min)
RC_EPAM14_2 517 (4.374) Cm (509:525)
1: Scan ES+
2.15e6
100
%
0
686.4
686.8
1029.3
1028.7
686.1
1029.6
685.9
1029.8
687.1
1030.2
685.6
687.3
1030.7
1028.2
1097.3
687.7
1097.8
710.2
1098.4
1105.3
680.7
710.7
711.4
522.9
585.8
1162.6
1240.9
400
600
800
1000
1200
1400
1600
m/z

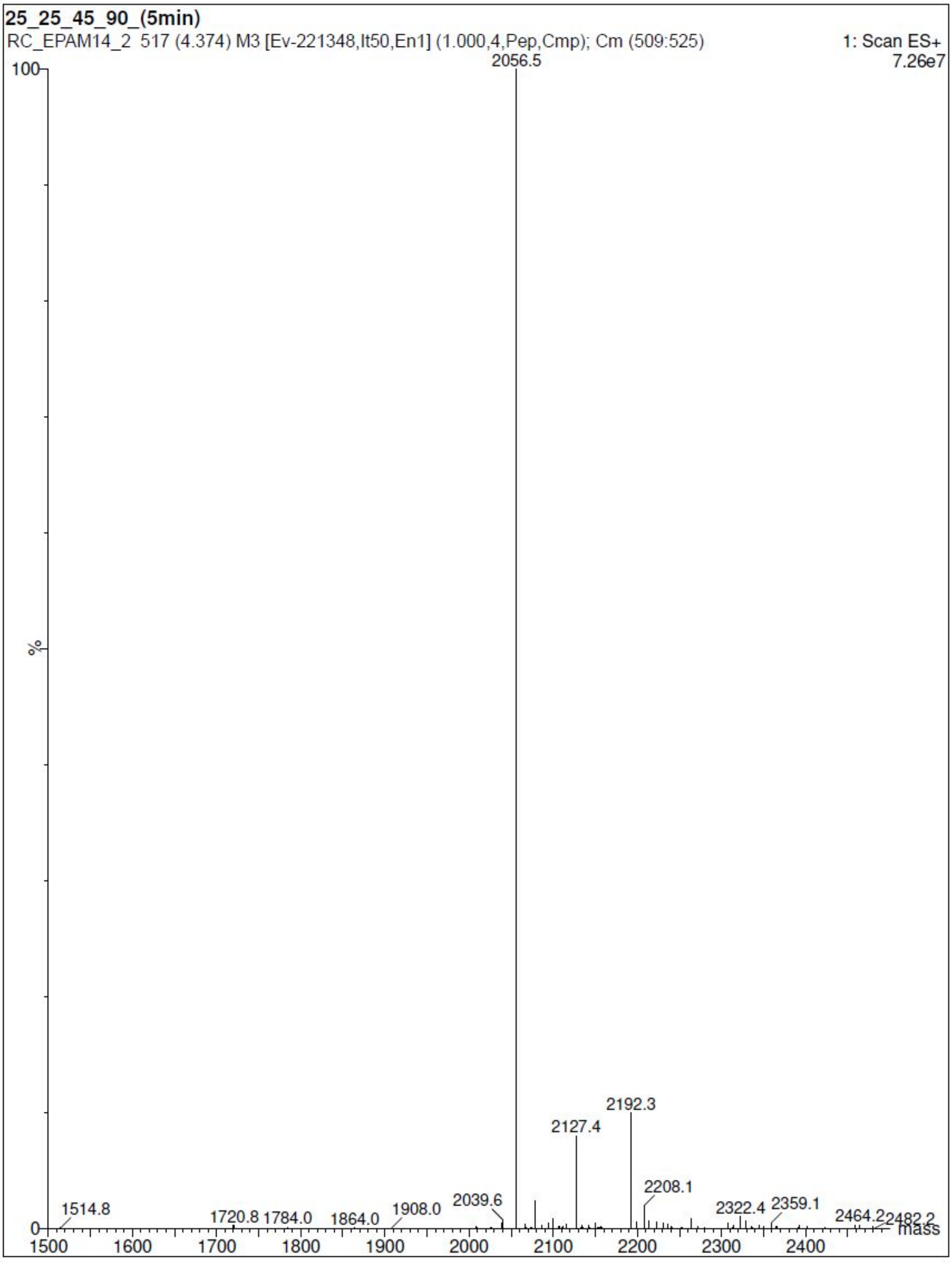
25_25_45_90_(5min)
RC_EPAM14_2 517 (4.374) M3 [Ev-221348,It50,En1] (1.000,4,Pep,Cmp); Cm (509:525)
1: Scan ES+
7.26e7
100
%
0
2056.5
2127.4
2192.3
2208.1
2039.6
1514.8
1720.8
1784.0
1864.0
1908.0
2322.4
2359.1
2464.2
2482.2
1500
1600
1700
1800
1900
2000
2100
2200
2300
2400
mass

RP-HPLC analytical trace of final bicyclic peptide

Injection volume: 20 mL of 1 mg/1 mL of water

Method: gradient of water/ACN + 0.1% TFA as below

wavelenght 275 nm

Time	Flow	%A	%B	%C	%D
0.01	1.00	5.0	0.0	95.0	0.0
5.00	1.00	5.0	0.0	95.0	0.0
20.00	1.00	90.0	0.0	10.0	0.0
25.00	1.00	90.0	0.0	10.0	0.0
28.00	1.00	5.0	0.0	95.0	0.0
33.00	1.00	5.0	0.0	95.0	0.0

A= ACN +0.1%TFA; C=Water +0.1%TFA

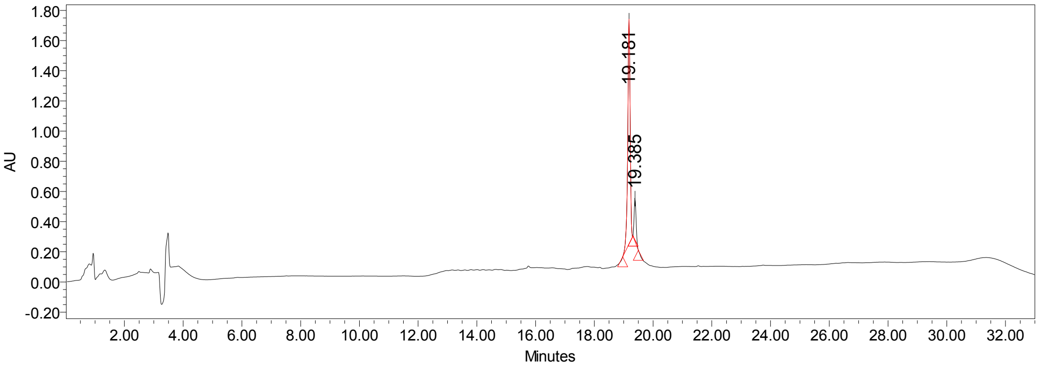

	Retention Time	Area	% Area	Height
1	19.181	9517274	95.37	1477216
2	19.385	4543729	4.63	282778

SUMMARY OF ANALYTICAL DATA FOR FINAL PEPTIDE

Analytical RP-HPLC	Mass signals Formula **C96H126N28O20S2** **e.m. 2055.9** **m/z**			
Rt (min)	$[M+H]^+$	$[M+2H]^{++}$	$[M+3H]^{+++}$	Rt (min) UPLC
19.18	2056.5	1094.3	686.4	4.33

Table S1. ^{1}H, ^{13}C, and ^{15}N resonances assigned for the peptide in solution.

Residue	***^{15}N / HN***	***$^{13}C\alpha$ / Hα***	***$^{13}C\beta$ / Hβ***	***^{13}C / H***
L-Pro1		61.7 / 4.47	30.5 / 2.03, 1.75	$^{13}C^{\gamma}$ 27.3 / $H^{\gamma}g$ 1.89, 1.64; $^{13}C^{\delta}$ 50.6 / H^{δ} 3.42, 3.29
Gln2	119.8 / 8.04	55.9 / 4.34	29.9 / 2.82, 2.68	$^{13}C^{\gamma}$ 34.1 / H^{γ} 2.32, 2.26; $^{15}N^{\varepsilon}$ 111.9 / H^{ε} 7.37, 6.79
Phe3	120.3 / 8.53	53.4 / 4.70		$^{13}C^{\delta}$ 131.8/ H^{δ} 7.17; $^{13}C^{\varepsilon}$ 131.6 / H^{ε} 7.31; $^{13}C^{z}$ 130.0 / H^{z} 7.28
Asn4				$^{15}N^{\delta}$ 111.8 / H^{δ} 7.46, 6.83
Cys5	121.4 / 8.29	57.7 / 4.71	43.3 / 3.12, 3.06	
Arg6	119.6 / 8.13	56.4 / 4.34	30.8 / 1.59, 1.52	$^{13}C^{\gamma}$ 27.0 / H^{γ} 1.33; $^{13}C^{\delta}$ 43.2 / H^{δ} 2.94
Trp7	126.1 / 8.15	54.4 / 5.04	30.1 / 3.09, 3.18	$^{13}C^{\delta1}$ 127.1 / $H^{\delta1}$ 7.16; $^{13}C^{\varepsilon3}$ 121.0 / $H^{\varepsilon3}$ 7.59; $^{13}C^{h2}$ 124.6 / H^{h2} 7.20; $^{13}C^{z2}$ 114.6 / H^{z2} 7.45; $^{13}C^{z3}$ 121.9 / H^{z3} 7.13
D-Pro8		63.4 / 4.44	32.1 / 2.18, 2.00	$^{13}C^{\gamma}$ 26.7 / H^{γ} 2.03, 1.97; $^{13}C^{\delta}$ 50.3 / H^{δ} 3.86, 3.61
L-Pro9		61.7 / 4.47	30.5 / 2.03, 1.75	$^{13}C^{\gamma}$ 27.3 / $H^{\gamma}g$ 1.89, 1.64; $^{13}C^{\delta}$ 50.6 / H^{δ} 3.42, 3.29
Gln10	119.8 / 8.04	55.9 / 4.34	29.9 / 2.82, 2.68	$^{13}C^{\gamma}$ 34.1 / H^{γ} 2.32, 2.26; $^{15}N^{\varepsilon}$ 111.9 / H^{ε} 7.37, 6.79
Phe11	120.3 / 8.53	53.4 / 4.70		$^{13}C^{\delta}$ 131.8/ H^{δ} 7.17; $^{13}C^{\varepsilon}$ 131.6 / H^{ε} 7.31; $^{13}C^{z}$ 130.0 / H^{z} 7.28
Asn12				$^{15}N^{\delta}$ 111.8 / H^{δ} 7.46, 6.83
Cys13	121.4 / 8.29	57.7 / 4.71	43.3 / 3.12, 3.06	
Arg14	119.6 / 8.13	56.4 / 4.34	30.8 / 1.59, 1.52	$^{13}C^{\gamma}$ 27.0 / H^{γ} 1.33; $^{13}C^{\delta}$ 43.2 / H^{δ} 2.94
Trp15	126.1 / 8.15	54.4 / 5.04	30.1 / 3.09, 3.18	$^{13}C^{\delta1}$ 127.1 / $H^{\delta1}$ 7.16; $^{13}C^{\varepsilon3}$ 121.0 / $H^{\varepsilon3}$ 7.59; $^{13}C^{h2}$ 124.6 / H^{h2} 7.20; $^{13}C^{z2}$ 114.6 / H^{z2} 7.45; $^{13}C^{z3}$ 121.9 / H^{z3} 7.13
D-Pro16		63.4 / 4.44	32.1 / 2.18, 2.00	$^{13}C^{\gamma}$ 26.7 / H^{γ} 2.03, 1.97; $^{13}C^{\delta}$ 50.3 / H^{δ} 3.86, 3.61

Table S2. NMR distance constraints and structural statistics for the ensemble of the 20 structures peptide.

NOESY distance constraints	
Intraresidue	30
Sequential *($\|i-j\| = 1$)*	21
Medium range *($\|i-j\| \leq 5$)*	3
Long range *($\|i-j\| > 5$)*	11
Hydrogen bonds	4
Torsion angles restraints[1]	
Backbone angles *($\varphi/\psi/\chi 1$)*	30
RMSD to main structure	
Backbone atoms region 2 .. 7, 10 .. 15 (Å)	0.41 ± 0.15
Heavy atoms region 2 .. 7, 10 .. 15 (Å)	1.56 ± 0.26
Ramachandran plot[2]	
Residues in most favored regions *(%)*	78.8
Residues in additionally allowed regions *(%)*	13.5
Residues in generously allowed regions (%)	7.7
Residues in disallowed regions (%)	0.0
RMS Z-score[3]	
Bond lengths *(Å)*	-1.891 ± 0.659
Bond angles	-3.085 ± 0.332
Dihedral angles	0.576 ± 0.239
Side chains planarity	-0.551 ± 0.200
Non-bonded interactions	-3.119 ± 0.245
Model quality	-3.792 ± 0.257

[1]Calculated with TALOSn (Shen & Bax, 2013); [2]Calculated with Procheck (Laskowski et al., 1996); [3]Calculated with WHAT IF (Vriend, 1990) included in Yasara software (Krieger et al., 2002).

Figure S1. Fragments of hetero-correlation spectra acquired for the peptide in solution. **A)** ^{1}H-^{15}N HSQC spectrum; **B)** Fragment of ^{1}H-^{13}C HSQC spectrum shown region aliphatic protons; **C)** Aromatic region of the ^{1}H-^{13}C HSQC spectrum. The resonance assignments shown as one-letter code, residue number, and name of ^{13}C atom.

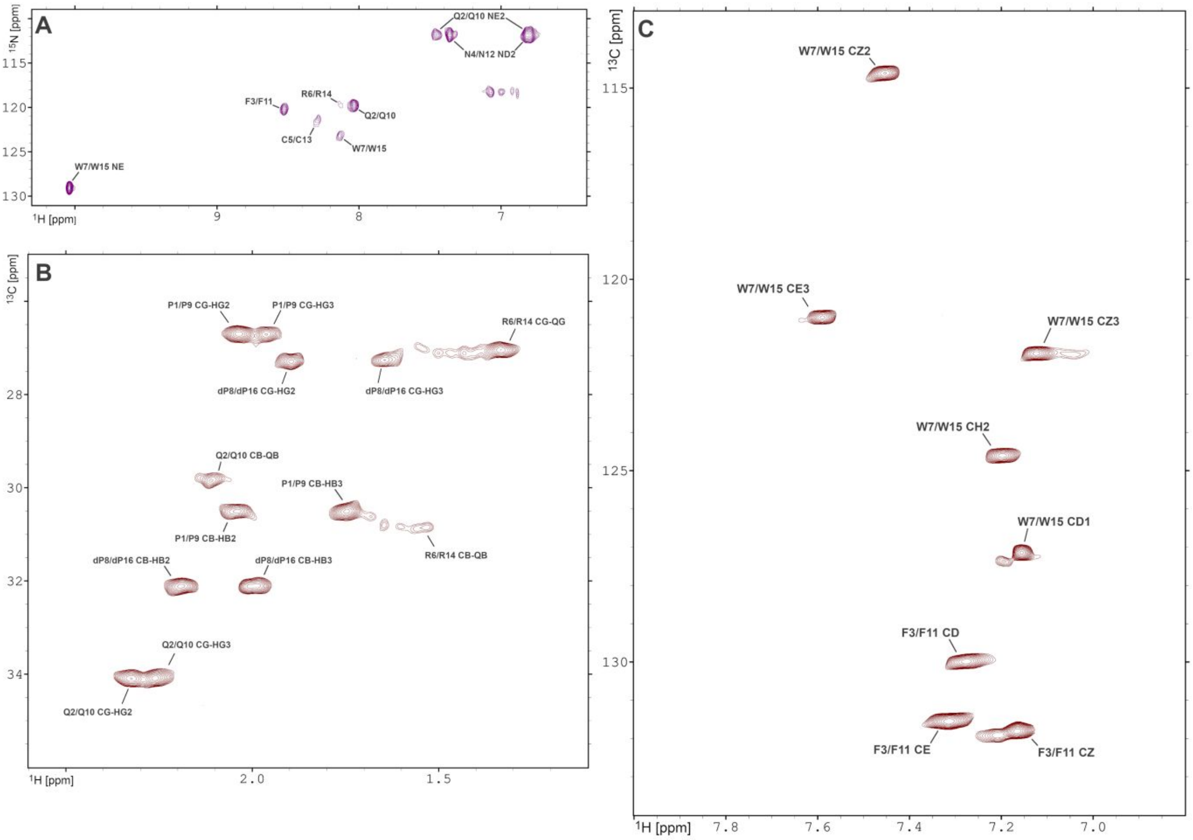

Figure S2. Superimposition of bicyclic peptide (cyano color), peptide **9** (green color) and peptide **10** (magenta color).

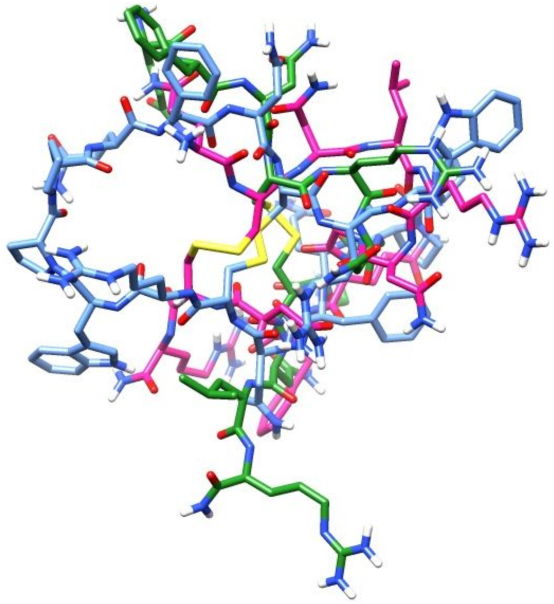

Figure S3. (A, left) crystal structure of DNA in complex with native arc repressor (PDB ID: 1PAR); (B) pose and interactions of the native arc β-sheet domain (crystallographic ligand) contacting ds-DNA (PDB ID: 1PAR); (C) superimposition of the docking poses of bicyclic peptide docked to TAGA DNA (sky blue) and CACA DNA (tan); (D) depiction of the interactions established between peptide and TAGA DNA; (E) depiction of the interactions established between peptide and CACA DNA. These images have been obtained with UCSF Chimera 1.16.

A

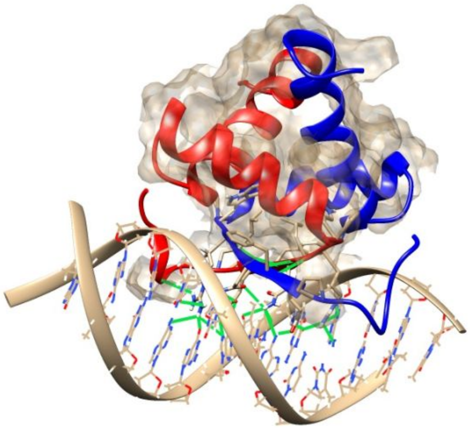

B

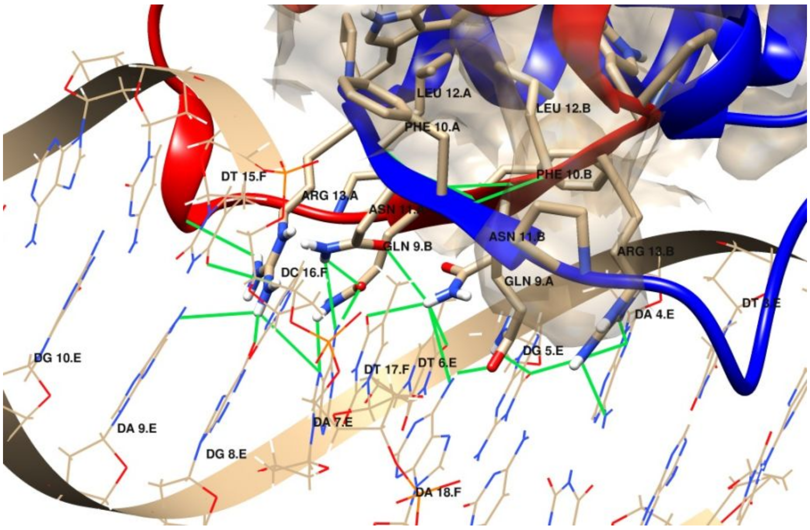

C

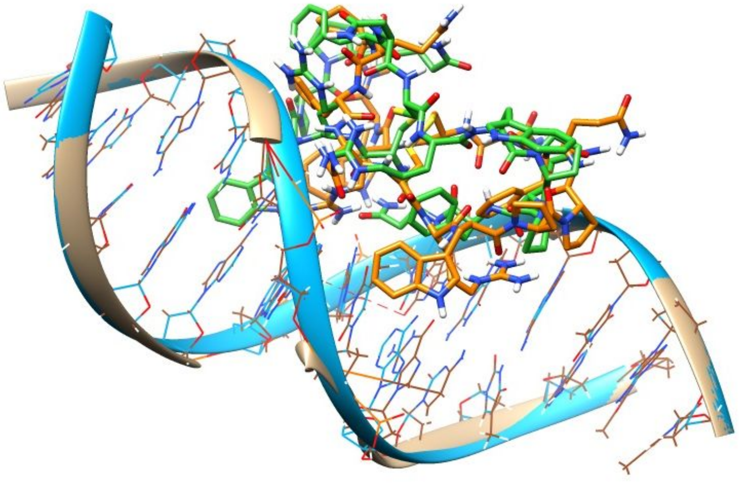

D

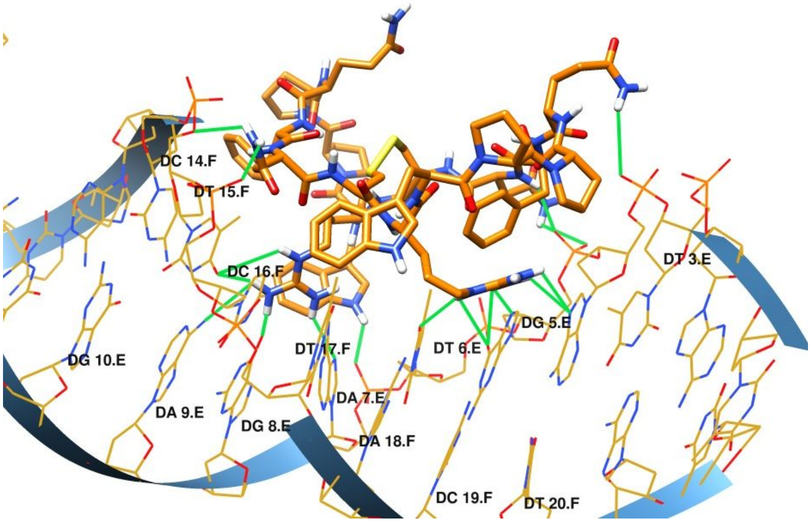
DC 14.F
DT 15.F
DC 16.F
DT 3.E
DG 5.E
DT 6.E
DT 17.F
DG 10.E
DA 7.E
DA 9.E
DG 8.E
DA 18.F
DC 19.F
DT 20.F

E

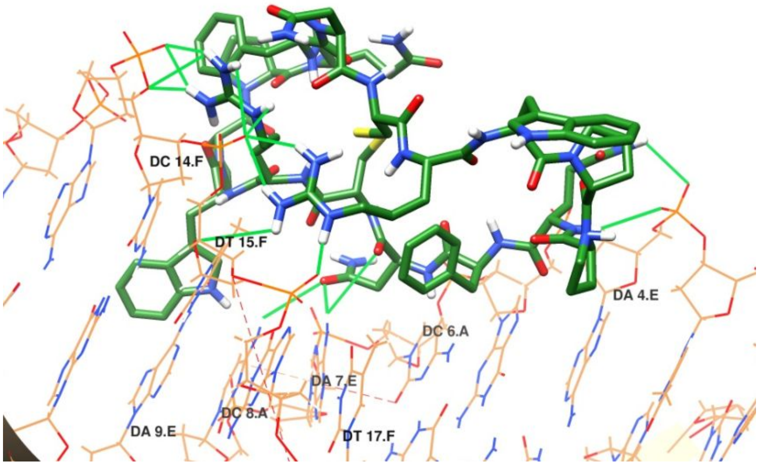
DC 14.F
DT 15.F
DA 4.E
DC 6.A
DA 7.E
DC 8.A
DA 9.E
DT 17.F

Table S3. DNA nucleotides from the crystal structure of the native arc β-sheet and the amino acid residues interacting with TAGA and CACA DNA sequences for both the co-crystallized ligand and the novel peptide analogue.

Letters E and F indicate the two ds-DNA chains. A and B indicate the two peptide (co-crystallized ligand or peptide) chains. The letters in parentheses next to each residue denote the nucleotide moiety with which the residue interacts: (A) adenine; (T) thymine; (G) guanine; (C) cytosine; (P) phosphate group.

**DNA nucleotides**	**Interacting residues**		
	**Co-crystallized ligand**	**peptide-TAGA**	**peptide-CACA**
DA 4.E	Arg 13.B	Gln 9.A (P), Arg 13.B(A)	Gln 9.A (P)
DG 5.E	Gln 9.A, Arg 13.B	Asn 11.A (P), Arg 13.B (G)	-
DT 6.E	Asn 11.B	Arg 13.B (T)	-
DA 7.E	Gln 9.B, Asn 11.A, Arg 13.A	Trp 14.A (P), Arg 13.A (A)	Asn 11.A (A)
DG 8.E	Arg 13.A	Arg 13.A (G)	Asn 11.A (mutated C)
DA 9.E	Arg 13.A	Arg 13.A (A)	-
DT 15.F	Arg 13.A	Arg 13.A (T)	Arg 13.A (P), Arg 13.B (P)
DT 17.F	Asn 11.B	-	-
DA 18.F	Gln 9.A, Asn 11.B	-	-
